# Asymptomatic coronavirus disease 2019 (COVID-19) in hospitalized patients

**DOI:** 10.1017/ice.2020.441

**Published:** 2020-08-26

**Authors:** Victor C. Passarelli, Klinger Faico-Filho, Luiz V. L. Moreira, Luciano Kleber de Souza Luna, Danielle D. Conte, Clarice Camargo, Ana H. Perosa, Nancy Bellei

**Affiliations:** 1 Infectious Diseases Division, Department of Medicine, Escola Paulista de Medicina - Universidade Federal de São Paulo, São Paulo, Brazil; 2 Instituto de Pesquisa PENSI - Sabará Hospital Infantil, São Paulo, Brazil

*To the Editor*—In 1 week, 9 in 120 asymptomatic inpatients (7.5%) were diagnosed with coronavirus disease 2019 (COVID-19) at a hospital with a universal face mask policy. The median length of stay was 11 days, suggesting nosocomial infections. Most were presymptomatic, with median cycle threshold value of 22, indicating high viral loads. Assessment of asymptomatic COVID-19 can help determine the true impact of the disease and improve knowledge on transmission potential, which is of paramount relevance for public health policies and for infection control.^1,2^

Between the July 6 and 12, 2020, 120 patients aged >18 years at São Paulo Hospital in São Paulo, Brazil, were screened for severe acute respiratory coronavirus virus 2 (SARS-CoV-2) with RT-PCR on nasopharyngeal specimens. All patients were assessed for symptoms (including fever ≥37.8°C, cough, anosmia, dysgeusia, dyspnea, myalgia, headache, and nasal discharge), and were asymptomatic at enrollment.

The hospital normally has ~600 beds, but during the pandemic, this hospital was divided between isolated COVID-19 units with 120 beds and general units with restricted capacity, leaving ~100 beds to non–COVID-19 patients. The hospital had a universal face mask policy for healthcare staff (surgical), patients, and visitors (cloth or surgical) at the time of sample collection. None of the patients were suspected COVID-19 cases nor had known exposure to confirmed cases, so the standard care for the condition which they were hospitalized was carried out normally for these patients. Outcomes were monitored until test results were received. If a test was positive, the patient was transferred to an isolated unit. This study was approved by the local ethics committee and all subjects signed a written informed consent form.

Data are summarized as percentages and medians (ranges), and 95% confidence intervals were calculated via the binomial method using free Statistics version 4.0 software.

## Results

Overall, 9 asymptomatic patients (7.5%; 95% CI, 3.48%–13.76%) were diagnosed with COVID-19 (Table 1). Two patients (22.2%) were in the hospital due to surgery, and the others were hospitalized due to clinical conditions. The median time of hospitalization was 11 days (range, 1–39).

**Table 1.** In-Hospital Demographic Characteristics and Outcomes for Each Patient

Patient No.	Age, y	Gender	Initial Cause of Hospitalization	Hospital Accommodation	Length of Stay When Tested, d	Time to Symptom Onset, d	Outcome
1	79	Female	Surgical, hip fracture	First floor, single bedroom	1	…	Discharge
2	53	Male	Surgical, aortic dissection	Third floor, single bedroom	39	…	Discharge
3	50	Male	Clinical, diabetic ketoacidosis	Third floor, single bedroom	11	…	Discharge
4	36	Female	Clinical, relapsing polychondritis	Third floor, shared bedroom (with patient 5)	11	2	MV/Death
5^a^	64	Female	Clinical, systemic lupus erythematosus	Third floor, shared bedroom (with patient 4)	15	3	SONC/ICU
6	81	Male	Clinical, urinary tract infection	Third floor, single bedroom	6	5	MV/Discharge
7	63	Male	Clinical, urinary tract infection	Tenth floor (same ward as patient 8), single bedroom	21	4	MV/Death
8	53	Female	Clinical, nephrotic syndrome	Tenth floor (same ward as patient 7), single bedroom	9	1	SONC/Discharge
9^a^	34	Male	Clinical, decompensated heart failure	Tenth floor, single bedroom	2	2	SONC

Note. ICU, intensive care unit; MV, mechanical ventilation; SONC, supplemental oxygen with nasal cannula.

a Patients with no definitive outcome and still in hospital when this manuscript was written.

All asymptomatic patients with COVID-19 had considerable comorbidities, including hypertension (n = 7, 77.7%), obesity (n = 5, 55.5%), and diabetes (n = 4, 44,4%). Also, 4 patients (44.4%) had immunocompromising conditions: 2 had rheumatic diseases, 1 had had a kidney transplant, and 1 had a nephrotic syndrome requiring high-doses of corticotherapy.

Notably, 6 patients (66.7%) developed respiratory tract symptoms a median of 2 days (range, 1–5) after the sample collection; thus, they were recategorized as presymptomatic at time of testing, and all required respiratory support: 3 patients (50%) required mechanical ventilation (of these, 2 died and 1 was discharged). The other 3 patients received supplemental oxygen with nasal cannula and 1 of them was discharged. The others are still in the hospital due to their comorbidities. The 3 asymptomatic patients were discharged without complications. The median cycle thresold (Ct) values were 22 (range, 19–37) and 38 (range, 35–39) for the presymptomatic and asymptomatic subgroups, respectively.

## Discussion

Most asymptomatic infections were detected in patients who had been in the hospital for longer than the median incubation period for SARS-CoV-2,^2^ which suggests nosocomial transmissions.

Considering potential exposure factors, patients 4 and 5 had shared a 2-bed room for at least 72 hours before they received positive test results from samples taken on the same day (Table 1). Notably, they were also among 5 others (patients 2–6) from this cohort who were on the same hospital floor when diagnosed. Despite their allocation to different wards, the same hospital staff circulate through all wards on a daily basis, suggesting a cluster.^2,3^

Our findings also suggest the underestimated potential of visitor contribution to viral spread in healthcare facilities. Patient 7 had received visits from his wife, who was asymptomatic and wore a face mask at the time, but a few days afterward developed symptoms and was indeed positive for SARS-CoV-2; therefore, she is retrospectively considered a possible source of exposure. Notwithstanding, patients 7 and 8 had been in the same ward for the previous 9 days and were diagnosed at the same time, regardless of being in different single bedrooms and using protective measures; we considered this to be another possible cluster.

Other factors might have imposed higher risk for these possible nosocomial transmissions. Universal masking may induce a false perception of protection that leads to neglect of other important protective measures,^4^ including social distancing and avoiding skin contact.^5^ The incorrect use of full protective personal equipment is also concerning, including inadequate hand hygiene^6^ or even the incorrect use of masks due to malpositioning, inadequate fabric materials, or inappropriate reuse. Scarce resources also play a part in nosocomial risk of transmission.^7^

Nevertheless, asymptomatic individuals, including patients and their visitors, may need to be considered when designing strategies to prevent outbreaks in the healthcare setting, regardless of a universal masking policy.

Finally, previous studies have suggested that only a small fraction of asymptomatic persons may eventually develop symptoms,^1–3,5^ but more than half of the initially asymptomatic patients in this study became symptomatic. Perhaps disruption of viral control seen in immunocompromising conditions from the presymptomatic subgroup influenced higher viral loads,^8^ demonstrated by the lower Ct values.^9^ However, previous reports have suggested similar potential for viral transmission in otherwise healthy, presymptomatic subjects as well,^3,5,10^ so it does raise concerns.

The inclusion of healthcare staff and visitors in the screening process could help improve knowledge on viral dynamics in this setting.

In summary, surveillance of asymptomatic COVID-19 in the healthcare setting may be an important measure in reducing nosocomial infections despite universal use of face masks, especially because presymptomatic patients may have high viral loads, suggesting the potential for transmission.
